# Book Reviews

**Published:** 1808-07

**Authors:** 


### CRITICAL ANALYSIS
### OF THE
### RECENT PUBLICATIONS
### ON THE
### DIFFERENT BRANCHES OF PHYSIC, SURGERY, AND MEDICAL PHILOSOPHY.

*Observations on the Inflammatory Affections of the Mucous Membrane of the Bronchiæ.* By Charles Badham, M. D. Member of Pembroke College, Oxford; Physician to his Royal Highness the Duke of Sussex, and to his Household; senior Physician to the Westminster General Dispensary; and Lecturer on the Practice of Physic. 12mo. London, 1808.

In the introductory chapter the learned author traces, with much diligence, the history of the early opinions concerning those complaints in which the breathing and secretion of the organs concerned in that process are particularly affected. In doing this he shows a nice discrimination on these important subjects, and proves that the ancients, with all the disadvantages under which they laboured from want of a knowledge of human morbid anatomy, were extremely judicious in their observations.

In the succeeding chapter we have a review of the "opinions of modern systematics on bronchial affections," or, as it might as well have been expressed, an account of the various descriptions of bronchial affections since the time that an attempt has been made to arrange diseases in nosological order. Hoffmann is allowed the credit of a most accurate description; and, indeed, the passage quoted by our author is almost as complete as if he had been aware of the causes of a disease, which, for want of sufficient opportunities of dissection, he imputes to a paralysis of the nerves belonging to the organs of respiration. The passage is so striking that we shall transcribe it from the work before us, with the author's note, and his remarks on Sydenham.

"Trahitur în hoc morbo summa cum difficultate et anxietate spiritus, et quiâ bronchiis, secedente a sanguine humore viscido seroso, repletis, nihil tamen sputi rejicitur aër admissus strepitum et ronchum in fistulis edit, donec præcluso penitus aëre, æger suffocetur. Quod antiquam evenit pulsus aliquot sæpe ante horis intermitit, sensîm gracilescit penitusque intercidit: non nunquam etiam mentis turbatio* cum extremorum frigore supervenit.— Med. Rat. Syst. sect. i. 17. xvi.

"The

---

* No writer but Hoffmann has remarked this symptom. It is evidently not a remark of the closet; the author has more than once been convinced of its accuracy. It is, however, only an occasional symptom.

"The description of a disease under the name of peripneumonia notha, which we owe to the illustrious Sydenham, has been regarded (perhaps erroneously) as an original account of an affection of the lungs not previously known. He appears rather to have described with greater felicity and correctness than others, an inflammatory affection of the bronchiæ; and one of the causes which may occasion the difference between his account and those of other writers, is probably this—that he describes the disease in its incipient state. The complaint is insidious; its first symptoms, therefore, would readily be overlooked by less accurate observers; whereas, in its advanced stage, with the symptoms of catarrhus suffocativus, nobody could fail to recognize it.

"Primo febris insulsu nunc incalescit æger nunc friget. Vertiginosus est: de capitis dolore quæritur lancinante quoties tussis importunius fatigat: urina turbida cernitur et rubens intensè: sanguis detractus pleuriticorum sanguinem refert. Anhelus sæpenumero spiritum crebro ac celeriter ducit; si moneatur ut tussim provocet, haud aliter dolet caput, ac sî in partes mox dissiliret. Dolet et thorax omnis, pulmonum, coarctatio auribus adstantium percipitur.

"In this description of Sydenham, several material symptoms are noticed, which belong chiefly to the first stage of the disease, and serve to mark its essential character. The state of the pulse, the peculiarity of the respiration, the appearances of the blood and of the urine, and the head-ach which attends, are all important particulars; and if the description itself were in any respect defective, if it had left us in doubt whether the affection were of an inflammatory nature or not, we should have our difficulties removed by adverting to the practical directions which follow. Venesection was employed, and even repeated, and an antiphlogistic treatment was adopted."

Lieutaud's account follows, which is shown not to differ materially: some just observations are made of the undefined term of *infarction*, adopted by this and most other writers before Cullen. In mentioning the last writer, Dr. Badham has fallen into the too common failing of most of his contemporaries. That Cullen was unequal to as much as he attempted cannot be doubted; that he followed Sydenham's descriptions whereever he could, is equally certain; and that what he added of his own now appears often unsatisfactory, must be admitted. But it is unnecessary to be always reminding us of this. We ought not to forget that he did much towards the destruction of the humoral pathology, that he taught us to study Sydenham, and that he directed us how to *aqcuire* that knowledge which a longer life than his could never have *imparted*.

In the third chapter the author attempts a subdivision of the diseases of these parts. This is introduced by some general remarks on the nature and effects of inflammation, according to the constitution, habits, and previous state of health in the subject. Taking the the term Bronchitis as a genus, it is divided into three species, acuta, asthenica, and chronica. The first of these is the disease which makes the subject of the work; the second the peripneumonia notha of Boerhaave and his followers; the third comprehends the humoral asthma of the same school, *tussis senilis*, and the varieties of each. We have many objections to the term by which our author designates the second division; perhaps, one may be, to the school to which it owes its origin. But such are our prejudices, that we should be much better pleased if the term chronica were used for any inflammation not acute; and for the last division, either the word habitual or periodical. We shall not, however, enter into a controversy about mere words, but proceed in the language of the author to examine the rest of his work.

Though it might seem more regular to begin with a description of the acute disease, yet Dr. B. very properly prefers first ascertaining, with as much precision as possible, the true character of a disease more frequently occurring, and tolerably well ascertained. By these means it will be more easy to mark the difference in the true diagnosis of the two complaints. The description of Bronchitis Asthenica is as correct as we might expect from so judicious a writer and teacher; we are much pleased with the concluding part, in which the reader is cautioned not to mistake the sequel of the disease for phthisis pulmonalis, how strong soever the resemblance may be in the emaciation of the patient, and even the appearance of the expectoration. It is suspected, with much probability, that most of the supposed cures of phthisis have owed their origin to this error. A proper attention is, therefore, given to the means of discriminating the two diseases; and with some judicious remarks on this subject this part of the work concludes.

The fourth chapter contains the history of Bronchitis acuta, with some cases and examinations after death. The first thing remarked of the disease is the suddenness of the attack, its violence, and its frequent termination at the end of a few days in extreme debility. After marking other more common symptoms to their termination, the author enumerates several which are more particularly striking and characteristic. The chief of these are the peculiar violence and suddenness, its earlier termination in death than most other pulmonary diseases, and the slowness of the pulse after the first inflammation has subsided. This is well illustrated by a few cases, the first of which we shall transcribe.

" About four years ago, a man of forty years of age, exceedingly muscular, and of health which knew no interruption, went out to Greenwich on Easter Monday, and heated himself by the coarse amusements which are customary on that occasion. He found himself ill in returning to town at night, went immediately to his bed, and was attacked with the symptoms above related. He was seen on the second day; it was then not too late to bleed him, and, as far as recollection serves, this operation was repeated, but with inconsiderable relief. Every other measure which seemed likely likely to be of service, was of course adopted; but he died within the week

"The chest was examined the day after. The bronchiæ were completely plugged up by a thick tenacious secretion; but the lungs were perfectly sound, and there were no adhesions, or other marks of disease."

Two other equally pointed cases follow, with the dissections; and the chapter concludes with some remarks on the probability that the secondary pectoral symptoms of measles and the dyspnœa, which supervenes to hooping cough, have usually their origin in this affection of the bronchiæ.

The fifth chapter contains the termination, pathology, and diagnosis of these diseases, and the sixth the treatment. In the first we have many very useful remarks, well illustrated by the writings of others; in the last a series of practical and judicious observations.

The work concludes with a chapter on the history and treatment of chronic coughs. We should here have been satisfied with the author's practical remarks, which, though not altogether new, are very judiciously chosen, without his theory concerning over excitement, consequent debility, and diminution in the activity of the smaller vessels.

An appendix contains two well related cases, with their dissections.

On the whole, we recommend this performance as a well written and useful εγχειριδιον, (the author will perceive our reason for preferring that word) and shall be always glad to see other single diseases taken up with the same diligence and judgment.

---

*An Exposition of the Practice of affusing Cold Water on the Surface of the Body, as a Remedy for the Cure of Fever; to which are added, Remarks on the Effects of Cold Drink, and of Gestation in the open Air, in certain Conditions of that Disease.* By Robert Jackson, M.D. Edinburgh. 1808.

For the present work, we are informed by a preface, we are indebted to a passsge in Dr. Currie's fourth edititon, in which Dr. J. is charged with arrogating to himself the merit of introducing the practice of affusing cold water on the surface of the body, as a remedy in fever. Dr. J. is further accused of having omitted the mention of Dr. Wright's name, to whom the discovery is said to be due. A note follows, in which Dr. Wright's claims are stated by the author of the present work. This we shall transcribe.

"Dr. Wright, the person alluded to in this place, was a practitioner in Jamaica prior to the year 1777: he stands in the Army List as surgeon for the 99th regiment, which was raised at a late period of the American war, and, I believe, sent to serve in the West Indies: he was appointed physician to the forces in the year year 1795; and, he lately filled the president's chair at the Royal College of Physicians of Edinburgh.—His claim to the discovery of affusing cold water on the surface is shortly this. Some buckets of cold sea water were dashed, at his own desire, upon his own person, while he suffered from fever during his passage from Jamaica to England in the year 1777. He published a statement of the fact in a periodical journal in the year 1786; but he did not then inform the public that he had repeated the experiment in the interval of nine years, between 1777 and 1786. If the experiment was not repeated in so long an interval, where we must suppose, from his official station, that opportunities for prosecuting the practice abounded, we cannot admit that he made a discovery; for, according to a just view of the subject, a medical discovery comprehends something more than a solitary experiment."

Dr. Jackson remarks, that the origin of the practice would have been of less consequence, had not his veracity been called in question; and should he be found wanting in so important a requisite, the authority of all his cases, observations, and whatever information he may offer, would be lost to mankind. To answer such a charge would have required only a statement of facts, which would have occupied a few sheets: but in examining the rest of Dr. Currie's reports it appeared, that an attempt was made to controvert the *principles* on which Dr. Jackson acted. This led to a reflection on the importance of a fair statement, not only on account of the author's reputation, but from the consideration of his duty to the public as an army practitioner Still further he conceives, that the principle assumed by Dr. Currie, and apparently established by the result of his correspondence, is simple enough to become *popular*, though in many respects erroneous. His own, on the contrary, he admits is more complicated, requiring the most minute examination, explained with difficulty, and not always within the comprehension of those who have even some previous medical science. He frankly acknowledges, that after the labour with which he has pursued the subject, he is still unable to produce an unerring rule by which the application of cold water may be safely regulated. The standard of heat, as ascertained by the thermometer, he conceives extremely fallacious. All this will be more minutely illustrated in the progress of the work.

The first part contains a short history of the introduction of cold bathing into medical practice as a remedy for fever. This is traced to the treatment of the Emperor Augustus, not omitting the hints contained in the writings of Hippocrates. From thence the practice is followed through what is left of the orientalists, and those travellers who have described their manners. It is not our intention to under value the labours of an author whom we have always treated with peculiar, but by no means unmerited, respect. We only regret that the mode of life he has chosen has prevented his application to those records which rarely occur but in extensive libraries. Had it been otherwise he would have met with a most interesting interesting paper by Dr. Armstrong, on the subject of cold bathing in fevers. That, in many respects, original author speaks of the practice as entirely in disuse; and, so contrary to the opinions of his contemporaries, that he leaves it to the consideration of the next generation. There is something at least prophetic in this; and we cannot help wishing that he had lived longer, and been sufficiently engaged in practice to have introduced so valuable a remedy. But to return to Dr. Jackson. The passage now before us offers so succinct an account of the controversy concerning the introduction of the cold affusion that we shall transcribe it, in order that we may confine our future remarks to the practical lessons with which the work abounds.

"I myself*, as appears by histories detailed in my treatise on the Fevers of Jamaica, published in the year 1791, employed cold bathing as a remedy for the cure of fever as early as the year 1774; and facts which are commonly known shew, that I introduced it into the hospitals of the British army at an early period of the war 1793. If the practice was known to some at that time, it was not yet commonly adopted. In my treatise on the Fevers of Jamaica, which was published in the year 1791, it is probable I may appear to arrogate to myself more of the claim of introducing the practice into notice than is justly my due; for I now know, what men acquainted with books knew then, that sponging or washing the body with cold water was employed by De Hahn for the cure of a malignant fever which ravaged Silesia in the year 1737. The remedy is mentioned by De Hahn in terms of strong commendation; but there are no grounds to believe that it obtained currency among medical practitioners in Germany, in consequence of what he said in its praise. The practice in fact does not appear to have made progress in Germany; and it was not recommended by any name of eminence in the British dominions in the year 1774, when I was led to bring an accidental suggestion to the test of experiment. The hint which led to this experiment was drawn from the conversation of Captain Cunningham, a seafaring man, who had been master of a transport ship at the siege of Havannah, and who was master of a merchant ship in 1774. I embarked with him as passenger for Jamaica in the beginning of the abovementioned year, and I recollect his having mentioned one day during the passage, when discoursing of the events of the war of 1756, the circumstance which I have related in my treatise on the Fevers of Jamaica respecting this subject, viz. that some sick persons who were on board of his ship, which appeared to have been a kind of hospital ship, threw themselves into the sea,—as if to drown themselves. The greater part of them, he observed, were recovered from the waves, and, as far as he recollected, they were restored to their senses, and

 benefited

* "Treatise on the Fevers of Jamaica, published 1791, notes, p. 48, &c."

benefited in their health by the ducking. The fact struck me strongly as applicable to the cure of fever; and, in consequence of this impression, I resolved to make trial of a similar experiment when a favourable opportunity should offer to me. This in fact soon presented itself, viz. in the latter end of May of the same year, in the person of a sailor, an European, lately arrived at Savanna la Mar, where I resided. This is the simple state of the case. I do not perceive that it is connected with circumstances which can afford any just grounds for the most scrupulous to suspect that it is not true; yet I have to remark that some have insinuated obscurely*, and others have asserted roundly, that its authenticity is doubtful†. If these or others expect legal proof, by the depositions of ocular witnesses, that I actually employed cold bathing for the cure of fever at the period stated in my treatise on the Fevers of Jamaica, I must confess that I cannot furnish them with such satisfaction; for it is more than probable that no ocular witness of the first experiment, and even of the subsequent repetitions of the practice during the period of my residence in that island, is now among the living. But though I cannot promise the satisfaction on this head that such persons may require, I believe the subjoined certificate from Dr. Gregory‡, Professor of Medicine

* "Medical Reports, vol. ii. p. 198: "*He (Dr. Jackson)* "*has unaccountably omitted to mention Dr. Wright's name and discovery, which was before the public, five years prior to the publication of his Treatise on the Fevers of Jamaica.*"

† "Edinburgh Critical Journal, April 1804.—This journalist observes, that "*if he (Dr. Jackson) employed the cold affusion so early as he pretends, the details of its effects do not, in all probability, redound very much to his credit; and these he has accordingly very prudently suppresed.*" The assertion is not true; the details of the practice, and its effects, are given at length in the book above alluded to; but it would be an insult to the public to take any further notice of the suspicions of this journalist, who appears to regard nothing less than truth and candour."

‡ "The fragment in Dr. Gregory's possession is an abstract from fuller notes made at different times respecting the history and cure of the Fevers of Jamaica, and it may be supposed to comprehend the sum of my experience on the subject at the time it was written. I did not know of its existence till the year 1798, when it was presented to me by Dr. Gregory. I thought it had been lost among some other papers which were lost in America; but I now find that it had been given to Dr. Cullen, or rather to Dr. Henry Cullen, not by myself, but by a person with whom I left it when I went to the continent in the year 1782*. The circumstance escaped his memory when I returned, and it did not come into my own recollection till lately. The fragment alluded to may thus be supposed to have been in Dr. Cullen's library at the time of his death, after which it, by some chance or other, came into the hands of a medical gentleman in Edinburgh, who gave it to Dr. Gregory, on hearing the Doctor refer in his class to some observa-

* "That it was in Dr. Cullen's possession, I conclude from his having mentioned my opinions on the Fevers of Jamaica in his class in the year 1787 or 1788."

Medicine in the University of Edinburgh, a person whom the editor of the Edinburgh Review himself will not suspect of collusion in the case, will be deemed conclusive evidence, in the opinion of most, that the remedy in question was recommended by me, and it

---

observations made by me on the connection of the moon with fevers, which were communicated to Sir Joseph Banks, to the best of my recollection, in the year 1787 or 1788. Such is the history of this fragment: it is now in the possession of Dr. Gregory, who has obligingly desired such passages as relate to cold bathing to be transcribed for my satisfaction. These, as private notes not intended to see the light, may be held to be satisfactory proof that I had witnessed the good effects of cold bathing in different forms of fever; and while they do this, they prove at the same time, that I did not consider it as a practice to be employed indiscriminately, or that I expected uniform success from it, without caution and previous preparation of subject. Its effects are first noticed in *remittent fevers*.—" In order to remove a topical pain, the evacuation (venesection) ought to be made as quick as possible; and it would be proper at the same time to place the patient *in semicupio*, and, perhaps, to apply cold, to dash cold water on the part from which we wanted to dislodge the pain, particularly if the cold could be brought to act on the part, as in the particular instance of topical affection of the head." &c. Page 44.

" *Malignant Fever*.—" The warm and cold baths, generous wines, lively conversation, and music, if they are applied with judgment," &c. Page 55.

" *Continued Fever*.—" It is an object of greater moment, if we know how to stop the progress of the disease at once, or to change its nature from dangerous to mild, and from continued to remittent. The cold bath, properly managed, promises fairly to attain this end. In the few cases where I have seen it tried, it produced the most amazing effects. It promoted sweat, procured rest, and gave such strength and vigour to the system, that a patient who before it was applied could not raise his head from the pillow without shaking and fainting, was able next day to walk about the room. In a word, it seems to be a remedy fit to do every thing that is wanted in this case, if the sensibility of the system be not very much impaired; and even then more may be expected from it than from any other we know. The water that was used was from the sea, and had a large proportion of salt dissolved in it. This is more particularly proper, where the sensibility is impaired. The warm bath too may sometimes be of signal use, particularly the alternate application of this and of the cold bath; but it requires great judgment to use them with full advantage; at the same time they appear to be in general innocent." Page 63

" *Yellow Fever*.—" For all those purposes the cold bath, particularly if a large portion of salt be dissolved in the water, is a most capital remedy. If the sensibility is not much impaired, we may expect to attain our end with certainty. If the sensibility is very much impaired, it is certainly doubtful, though it promises more than any thing I know. There are a great many circumstances, however, which must go along with the use of it, in order to give it full power, and every advantage, particularly the prudent use of the warm bath and of wine," &c. Page 69.

" Transcribed by Mr. John Thomson, from a fragment of Dr. Jackson's original papers, which have been long in my possession, and forwarded by me to Dr. William Robertson of Bath.

(Signed) " J. GREGORY."

*Edinburgh, Oct.* 24, 1807.

it may thence be presumed, was employed by me at a date prior to the appearance of Dr. Wright's narrative, probably prior to the existence of his experience; for the fragment from which the extract is made was written in Jamaica, and necessarily written not later than the year 1777, as I left the West Indies early in 1778 to serve with the King's troops in America. The narrative by Dr. Wright, which forms the ground-work of Dr. Currie's volumes on the effects of water, made its appearance in the year 1786; my treatise on the Fevers of Jamaica was not published till the year 1791, consequently I might have known of it; but it does not follow, as the editor of the Edinburgh Review arrogantly pronounces, "that I could not be, or ought not to have been ignorant of it." I might address similar language to others; but it is not language to be addressed to a gentleman. My treatise on the Fevers of Jamaica was published in the year 1791. It contained stronger commendation, and fuller proof of the benefits of cold affusion, in certain circumstances of fever than any modern book then extant. It was reviewed in most of the periodical journals of the time, and it did not, perhaps, stand lower in their estimation than the narrative of Dr. Wright, which is now held to be so important. This is the fact; yet, I do not therefore say or insinuate that Dr. Currie acted unaccountably or disingenuously in not referring to it in the work which he published in 1797, six years after it made its appearance; I only contend that, as Dr. Currie derived no hints of information from my treatise of 1791, my claim to credence is not inferior, when I aver that I derived nothing from Dr. Wright's narrative of 1786. The fact is, I did not know of it; and if I did not know of it, it is plain I could not notice it. That is clear; but I now add, that had I then been acquainted with it, and taken all the light from it which it can be supposed to give, the task of making a book from such meagre materials, without some experience of my own, would have far exceeded my abilities. The instance of the success is solitary, or next to solitary; and when I see that its author, whom I allow to be a zealous physician, made no use of his discovery in the period of the nine years intervening between 1777 and 1786, though we are given to understand by the author of the Medical Reports that he had been in a foreign country in the interval, and, it would further appear, employed in some station of public service, where he could not be supposed to be without opportunities, indeed where occasions must have necessarily abounded which called for the most powerful aids of the medical art, the narrative sinks to the bare record of a fact: which, however important in itself, led to no consequences in the hands of its recorder, and cannot therefore be fairly supposed to be calculated to form a basis on which another could establish a system of practice."

The author proceeds next to detail his further experience. This was in the 3d Regiment of Foot or Buff, to which he was, in 1793, appointed regimental surgeon, as the only road by which, according to Mr. Hunter's regulations, he could arrive at the office of army army physician. Here our author had ample opportunity of tracing all the varieties of what he calls infectious fever, usually denominated Typhus. The regiment had received a number of recruits, and among them the disease could scarcely fail to be introduced. The attention to bathing and athletic exercises prevented its extension. But in the summer of 1794, Jersey being fixed upon as a depot for organizing and disciplining new recruits, sickness became very general, and the infectious fever of jails or hospitals attacked them in their crowded quarters. The 88th Regiment arrived at this time, and their surgeon, Mr. M'Gregor, was attacked with fever. During this time Mr. Thompson, then assistant surgeon of the Buffs (Dr. Jackson's regiment) was ordered to attend the hospital of the 88th. Mr. Thompson having seen the advantages attending cold affusion in the treatment of the Buffs, adopted it on the present occasion. Dr. M'Gregor acknowledges the advantage of it in his Medical Sketches, as well as in his correspondence with Dr. Currie.

In the autumn of 1794, the Buffs arrived at Bergen-op-Zoom, in high health, but very early in the autumn the usual fever attacked most of them, excepting only the commissioned officers. The cold of 1794 and 1795 was unusually severe, and the quarters of the troops were, for the most part, barns, without the aid of artificial heat. Even under these apparently unfavorable circumstances Dr. Jackson was not deterred from his favorite remedy. His plan was, however, in the first instance, to rub the skin well with a brush, cleansing it at the same time with soap and warm water. The water being intensely cold, affusions were not always attempted. Sponging, or even sprinkling, were found sufficient. Under this treatment the mortality was inconsiderable, compared with the extent and severity of the disease.

The division of troops under the command of Sir Ralph Abercrombie, in the year 1795, rendezvoused at the Cove of Cork, in the month of October. Being detained the greater part of the winter, and in very confined quarters, the mortality was considerable from the usual causes. That division which was under the care of our author at a temporary hospital, in Spike Island, was treated with cold affusion with great success. Even in the hospital ship John, which conveyed the sick and convalescents to the West Indies, the same mode of treatment, varied according to circumstances, was adopted in every stage of the disease, for the most part with advantage. The author's particular situation enabled him to form a fair estimate of the expectations to be formed of the remedy in the more advanced period of fever. Here he often found himself disappointed. At the Mole, in St. Domingo, he assumed the office of surgeon of the 56th. Regiment for a period of six weeks, in order to ascertain, with some certainty, how far the dreaded fever of that climate might be subdued by any mode of treatment. Under certain conditions of fever he found the greatest advantages from cold affusion, but soon perceived that it was not

 to to be indiscriminately applied, and that in many instances it was necessary to prepare the subject by bleeding and other means.

In the year 1799 the Russian auxiliary forces, being ordered to Jersey and Guernsey, and crowded in the transports during boisterous weather, became sickly. Dr. Jackson having the medical superintendance committed to his care, and recollecting the well-known custom among those people of submitting themselves to the extremes of heat and cold, conceived the present a fair occasion to try the effects of a similar practice in the treatment of fever. Unsanctioned, however, as it was by the German practitioners, who form the principal medical assistants in a Russian army, the trial could be only partial. In the cases in which it was employed it was found perfectly safe, and often advantageous. In the following year Dr. Jackson was appointed army physician, and head of the depot at Chatham. Here he had ample opportunities of ascertaining the full effect of his favorite remedy, and the success was demonstrated by the army returns of sick and cured. When the depot was removed to the Isle of Wight, the accommodations were inadequate to the necessities of the sick, the list of which increased during the whole winter. The disease was in its highest state of aggravation, yet the returns show that the balance of success was in favour of the warm and cold affusions.

" As this is proved," says our author, " by evidence which cannot be doubted, it must appear to the reader to be a striking, though it is not a singular instance of the caprice of medical opinion, that cold affusion for the cure of fever should be hailed as a discovery of great importance to the interest of the British army, and as such thrown under the protection of the Commander in Chief* in the year 1805, while it was represented, in the year 1801, by the present Physician-General † to the same Commander in Chief, as one of the dangerous and destructive practices of the author of this Exposition."

The second part begins with a vindication of the author's publication on fevers. In this he explains how he has been misunderstood by Dr. Currie; a subject on which we have before had occasion to remark. The principles of the two are contrasted, to show not so much the superiority of the author's own, as that, long before the publication of Dr Currie's work, he had systematically arranged his plan of operations, and on a mode of reasoning totally unconnected with the theory or opinions of Dr. Currie. On this subject we say as little as possible; first, because it has already been before us; next, because Dr. Currie is no longer living to vindicate himself; but most of all, because his friends, who are very numerous, and the partizans of his opinions who are still more so, are not likely to be convinced by what we can say; and those

* " Dedication of the 4th edition of Dr. Currie's Medical Repo

† " Remarks on the Medical Constitution, &c. P. 122."

those who have impartially attended to the controversy need no information from us. Passing over, therefore, the expressions quoted from Dr. Currie, which his best friends will wish had been omitted, we shall just notice the pointed manner in which Dr. Jackson remarks the different opinions and different mode of applying this powerful remedy, by Dr. Currie and himself. With Dr. Currie he remarks, the fundamental, if not the indispensable, conditions which warrant the safety and insure the success of the effect of cold affusion on the surface are increased heat and high temperature. The reduction of temperature, or subtraction of heat, by the application of cold water, is maintained to be the cause which dissolves the febrile association, and thereby cures the disease. When the presence of heat is indicated by the thermometer, the remedy is applied without restriction, and with equal decision is forbidden when the instrument marks a reduced heat. Dr. Jackson, on the contrary, conceives the bare circumstance of temperature extremely fallacious; every concomitant symptom is with him important. The heat he determines by sensation; but in all those cases in which any particular organ is affected by inflammation or congestion, or even where the actions, by which life is supported, seem too languid to be consistent with the due functions of the organs, evacuation was premised in a degree proportioned to the symptoms and the effect produced. If the skin was cold and insensible, the warmth and sensibility were roused by warm bathing and friction.

We have next a comparative review of the success of this practice, under our author, and the authority of other writers on the same subject. A long note is annexed on the propriety of some arrangement in the West Indies, by which newly arrived medical men may be apprised of the peculiarities of climate, and its influence on those who are consigned to military employments. This part of the work concludes with a further vindication of the author from Dr. Currie and the Edinburgh Reviewer.

[ To be concluded in our next. ]

